# PMMA Third-Body Wear after Unicondylar Knee Arthroplasty Decuples the UHMWPE Wear Particle Generation *In Vitro*

**DOI:** 10.1155/2015/575849

**Published:** 2015-03-19

**Authors:** Alexander Christoph Paulus, Manja Franke, Michael Kraxenberger, Christian Schröder, Volkmar Jansson, Sandra Utzschneider

**Affiliations:** Department of Orthopedic Surgery, University Hospital of Munich (LMU), Campus Großhadern, Marchioninistraße 15, 81377 Munich, Germany

## Abstract

*Introduction*. Overlooked polymethylmethacrylate after unicondylar knee arthroplasty can be a potential problem, since this might influence the generated wear particle size and morphology. The aim of this study was the analysis of polyethylene wear in a knee wear simulator for changes in size, morphology, and particle number after the addition of third-bodies. *Material and Methods*. Fixed bearing unicondylar knee prostheses (UKA) were tested in a knee simulator for 5.0 million cycles. Following bone particles were added for 1.5 million cycles, followed by 1.5 million cycles with PMMA particles. A particle analysis by scanning electron microscopy of the lubricant after the cycles was performed. Size and morphology of the generated wear were characterized. Further, the number of particles per 1 million cycles was calculated for each group. *Results*. The particles of all groups were similar in size and shape. The number of particles in the PMMA group showed 10-fold higher values than in the bone and control group (PMMA: 10.251 × 10^12^; bone: 1.145 × 10^12^; control: 1.804 × 10^12^). *Conclusion*. The addition of bone or PMMA particles in terms of a third-body wear results in no change of particle size and morphology. PMMA third-bodies generated tenfold elevated particle numbers. This could favor an early aseptic loosening.

## 1. Introduction

Unicondylar knee arthroplasties (UKA) in the meanwhile show excellent results in the treatment of a medial compartment osteoarthritis, which is certainly due to the sparing of soft tissues that results in better tibiofemoral and patellofemoral kinematics [1] and an increased range of motion compared to total knee arthroplasty (TKA) [2]. While the 10-year-survival rates of UKAs have been shown to be equivalent to those of modern TKA [3], clinical evidence has demonstrated a higher revision rate of UKA compared to TKA [4]. It is commonly accepted that accumulating wear debris after total joint arthroplasty finally leads to an aseptic loosening of the prosthesis, although the exact mechanism of this inflammatory process is not understood in detail yet [5, 6]. But in several studies it could be demonstrated that number, size, and shape of the wear particles influence the extent of the inflammatory biological reaction resulting in periprosthetic osteolysis [7–9]. Submicron particles, especially, increase the biological reaction [8]. It can be assumed that even minor changes in the wear rate have distinctive effects on the amount of accumulating wear particles. This correlation between the gravimetric wear rate of tibial inserts in knee simulator tests and the particle number analysis could be shown in a previous study, as minor changes in the particle size or wear rate showed considerable effects on the particle number [10]. Third-body wear after UKA, especially, might influence the wear generation and the particle morphology and thus lead to an early failure of the prosthesis. Bone and cement fragments (polymethylmethacrylate, PMMA) that occur during the implantation of the prosthesis can easily be missed in the posterior regions of the femorotibial joint gap relating to the minimally invasive approaches [11].

The aim of this study was to analyze the influence of third-bodies (bone- and PMMA-particles) on number, size, and shape of wear debris generated in an UKA joint simulator. First it was hypothesized that an occurrence of third-body debris can lead to elevated particle numbers. The second hypothesis was that bone as well as PMMA debris alters size and shape of the generated particles.

## 2. Materials and Methods

The simulator experiments have been described in a previously published study [12]. Sections 2.1–2.3 summarize the simulator tests.

### 2.1. Prostheses

For this investigation, fixed bearing unicompartmental knee prostheses (Univation-F, Aesculap, Tuttlingen, Germany) were used with a metal on polyethylene articulation.

The intermediate-sized femoral and tibial (F3/T4) components were made from CoCr29Mo6 alloy and the gliding inserts were composed of UHMWPE (GUR 1020, *γ*-irradiated, 30 ± 2 kGy). The inserts were fixed at the tibial baseplate component by a snap-fit-mechanism. The medial side of the meniscal bearing had a concave shape and the lateral side was planar. Before testing, the bearings were accelerated aged, according to the standard as described in the “ASTM F2003 - 02(2008)” to simulate the oxidation process of UHMWPE in air [14].

### 2.2. Simulator Specifications

Before wear testing the gliding surfaces were preconditioned in the test solution until no increase of weight was measurable. The test fluid simulated synovial liquid with a protein content of 30 g/L. The applied lubricant was changed every six days (25% (v/v) newborn calf serum (Biochrom, Germany) with 0.1% (m/v) sodium azide solution in sterile water with EDTA (AppliChem, Germany) for pH stability and Amphotericin B (Biochrom, Germany) as a fungicidal). The lubricant was changed every 0.5 million cycles. The specimens were tested on a servohydraulic knee wear simulator (EndoLab, Germany) with four test stations; the test specifications followed the ISO [15].

### 2.3. Wear Particle Generation

The simulator was stopped at 8.0 million cycles; the overall test period was divided into three parts. The first part was a standard test with 5.0 million cycles as prescribed by the ISO [15]. In the following two periods, the test solution was contaminated with third-body wear debris in a concentration of 5.0 g/L. The particles were produced by a micro-bone-mill (Aesculap GB060R, Tuttlingen, Germany). Between 5.0 and 6.5 million cycles cortical porcine bone particles were added to the test lubricant. From 6.5 to 8.0 million cycles, cement particles with zirconium dioxide as radiolucent (Palacos R, Heraeus Medical, Germany) were mixed within the simulator lubricant. The test phase after adding the different particles was therefore during the steady state phase of the inserts [12]. The morphologic parameters of the debris can be found in Table 1. Mean diameter (MD), equivalent circle diameter (ECD), form factor (FF), aspect ratio (AR), and roundness (R) of the third-body wear debris were recorded [16].

### 2.4. Particle Isolation

Within every 500000 cycles the simulator was stopped; the serum was removed and digested in the following using the “acid digestion” method [17]. The digested lubricant was then centrifuged at 5000 g for 30 minutes to remove the third-body wear debris (the used bone particles consist of milled cortical bone, which has a density of ≈1.5 g/cm^3^ [18]; the PMMA particles had a mean density of ≈1.18 g/cm^3^; the UHMWPE particles had a density of <0.96 g/cm^3^). The specimen was taken out with a pipette (Gilson, Pipetman, made in France, GD29041) three times 1 mL, while the pipette tip was dunked for about 1 cm in the centre of the Falcon tube. 10 mL of each serum sample supernatant was added to 50 mL of hydrochloric acid (37% v/v; Merck, Darmstadt, Germany) and mixed with a magnetic stir bar at 60°C for approximately one hour. Then, 3 mL of this digestion solution was added to 150 mL of methanol (Merck, Darmstadt, Germany) and filtered through a 0.02 *μ*m polycarbonate membrane (Anodisc 47, Whatman plc, Maidstone, Kent, United Kingdom). The filter membrane was then dried for 6 hours and sputter-coated with gold.

### 2.5. Particle Analysis

The particles recovered on the filter membranes were imaged by scanning electron microscopy (SEM, Zeiss EVO, Carl Zeiss Microscopy GmbH, Jena, Germany). The particles were analyzed at a magnification of 5000–10000 diameters. 20 random, nonoverlapping fields of view were analyzed per sample. Images of each field of view were captured, and the particles were measured using a digital image analysis program [10] (Leica QWin, Image processing and analysis application, Leica Microsystems, Wetzlar, Germany).

The boundary of each particle was defined on the basis of a gray-scale level threshold.

In accordance with the ISO, mean diameter (MD), equivalent circle diameter (ECD), form factor (FF), aspect ratio (AR), and roundness (R) were recorded [16]. According to Sieving et al. [13] the percentage of particles with an AR in the range from 1 to 2.39 and ≥2.4 was calculated. Furthermore, the particle number was calculated using the following formula as reported before (*N*_(*p*)_ is absolute particle number; *n*_(*p*)_ is examined particles; *G*_(*v*)_ is volumetric wear rate; *d*_(*m*)_ is equivalent circle diameter) [10]:

(1) $$\begin{array}{c} N_{\left(p\right)}=\frac{n_{\left(p\right)}\times G_{\left(v\right)}}{\sum_{k=1}^{n}\left(\left(\pi \times d_{\left(m\right)}^{3}\right)/6\right)}. \end{array}$$

The mentioned wear rate was recently found and described by Schroeder et al. in a recently published manuscript [12]. The numbers were calculated from the mean volumetric wear rate values.

### 2.6. Statistical Analysis

Statistical analysis was performed to analyze the significance of variance between the three groups using the Kruskal-Wallis one-way analysis of variance by ranks, as the data showed a nonparametric distribution. The differences were considered significant at *P* values < 0.05.

## 3. Results

### 3.1. Wear Particle Size and Morphology

Overall the particles of all groups showed similar size distributions of polyethylene wear, with a rounded median ECD of 0.13 *μ*m (min: 0.06 *μ*m; max: 3.38 *μ*m) for the bone group, 0.12 *μ*m (min: 0.06 *μ*m; max: 3.27 *μ*m) for the PMMA group, and 0.12 *μ*m (min: 0.06 *μ*m; max: 2.92 *μ*m) for the control group. All differences were statistically significant (*P* < 0.05).

**Figure 1:**
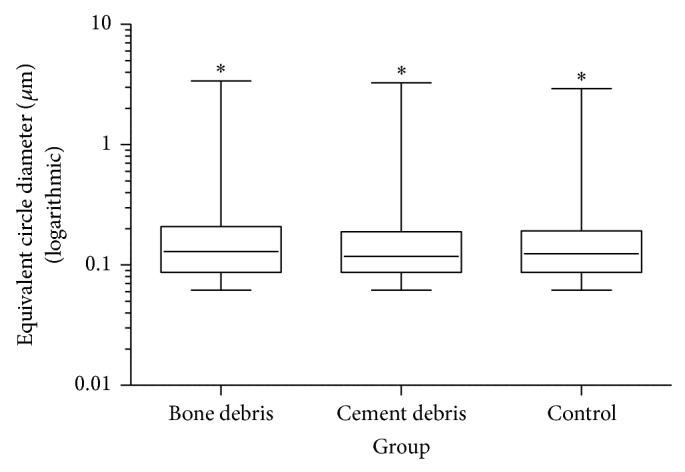
Size distribution measured by the ECD of all groups in a logarithmic box plot. The results of all groups differ significantly (^*^ *P* < 0.05).

**Figure 2:**
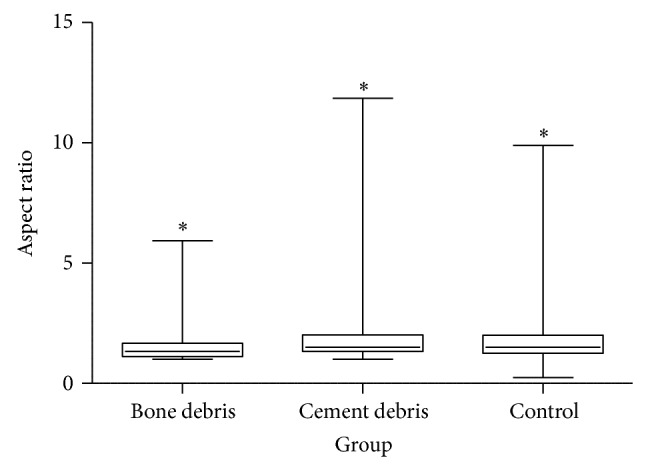
The morphology of the particles given by the aspect ratio. The cement debris group tends towards more fibrillar particles. The results of all groups differ significantly (^*^ *P* < 0.05).

Furthermore, the majority of all analyzed particles were approximately round in shape, smooth, granular, and irregular and had similar AR values (median): 1.34 (min: 1.00; max: 5.93) for the bone particle group, 1.51 (min: 1.00; max: 11.86) for the PMMA particle group, and 1.51 (min: 1.00; max: 9.90) for the control group. All size and shape parameters can be found in Table 2 (statistically significant results, *P* < 0.05). The particle size distribution is demonstrated in Figures 1 and 2 using box plots.

99.77% of the particles in the bone group were <1.0 *μ*m, 99.61% in the PMMA group and 99.71% in the control group were in the submicron size range.

**Figure 3:**
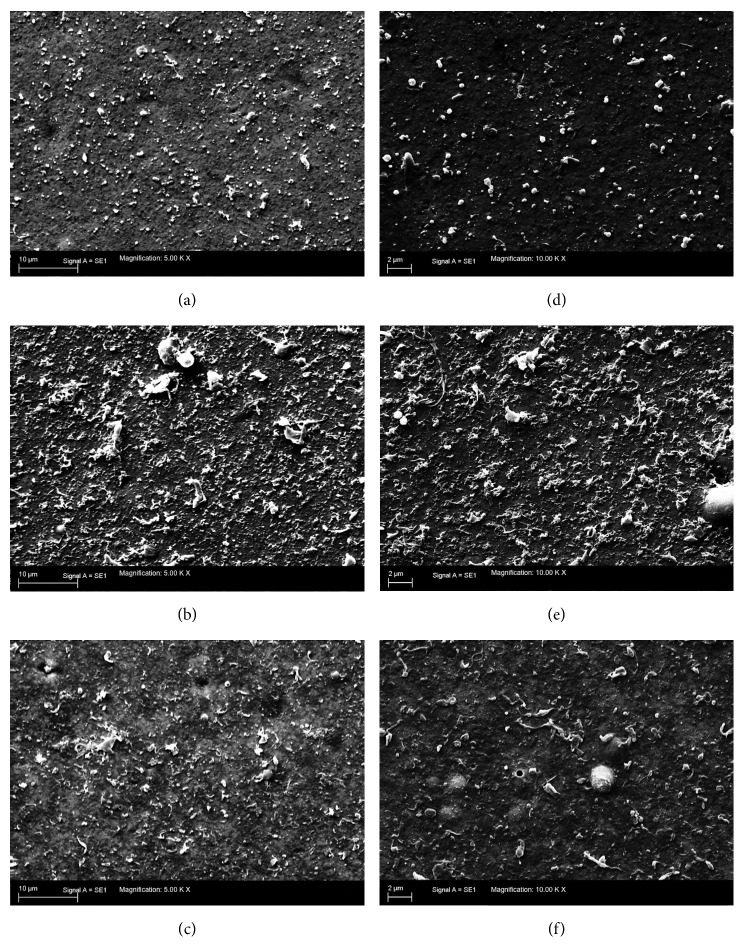
SEM sample images of all tested groups. (a) and (d) show the debris of the bone debris group; (b) and (e) demonstrate the enormous number of particles in the cement group; (c) and (f) serve as examples for the control group. (a), (b) and (c) are 5000x magnified; (d), (e) and (f) are 10000x magnified.

**Figure 4:**
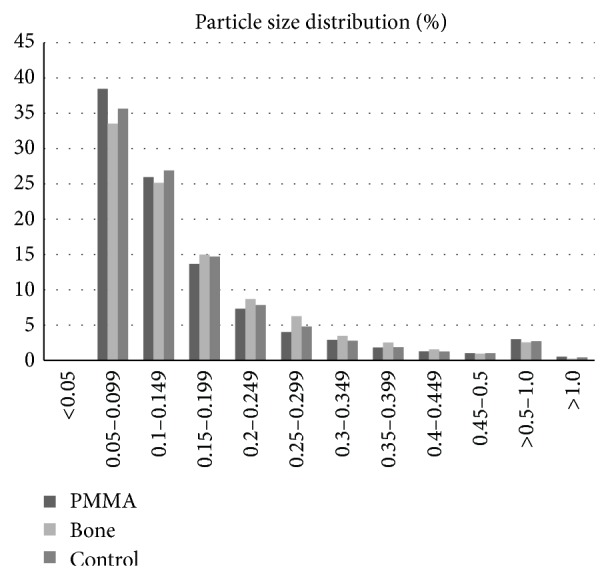
Particle size distribution measured by the ECD for each size interval.

In the bone particle group 6.35%, in the PMMA group 13.42%, and in the control group 9.84% of the particles had an AR ≥ 2.4 (Table 3).  Figure 3 shows example SEM images of the wear particles and gives the impression of mainly round and granular particles. Furthermore, a particle size distribution for each size interval is given in Figure 4.

### 3.2. Number of Particles

**Figure 5:**
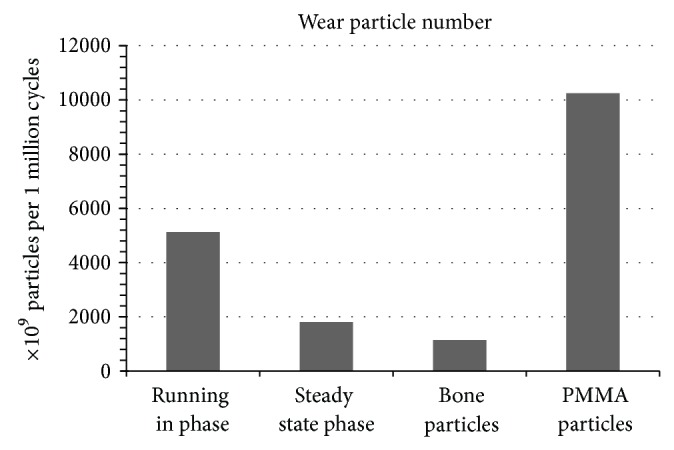
The change of the polyethylene wear particle number in the progress of the simulator tests, respectively after the addition of the third-body wear.

We found differing particle numbers for each group. First, a difference between the running in and the steady state phase was found. In the running in phase 5.126 × 10^12^ particles were calculated per 1 million cycles. In the steady state phase the particle number decreased to 1.804 × 10 10^12^. Interestingly, the addition of bone particles did not lead to an increase of the particle number (1.146 × 10^12^), whereas the added PMMA particles in the PMMA particle group decupled the polyethylene wear particle number (10.252 × 10^12^; Figure 5). The huge number of accumulating particles can already be suggested in Figures 3(b) and 3(e).

## 4. Discussion

This study demonstrated that free cement debris can significantly increase the generation of wear particles in unicondylar arthroplasty. The first hypothesis could only partly be accepted, as only the addition of PMMA third-body wear debris lead to higher particle numbers, whereas cortical bone particles did not affect the particle generation. The second hypothesis had to be rejected; the particle morphology was not altered by third-body wear debris clearly, although statistical tests showed highly significant differences.

Clinical evidence as well as retrieval studies had disclosed the issue concerning third-bodies after UKA [11, 19]. Schroeder et al. had proven in a simulator-based study that the wear rate is definitely influenced by cement third-body wear debris [12]. But so far, there are no studies known by the authors concerning the influence of third-body wear in UKA on the generated particle morphology and number. It is basically known that particle size, morphology, and number affect the biologic response resulting in an osteolysis that finally leads to an aseptic loosening and consequently to failure of the prosthesis [7, 8]. Therefore, our objective was to analyze the generated wear particles of a fixed bearing UKA under the influence of third-body debris like cortical bone and cement in terms of particle size, morphology, and number.

Schroeder et al. previously described the simulator testing [12] that defined a high contamination of third-body wear particles of 5.0 g/L, as former studies have shown a negligible effect of third-bodies in a concentration of 3.0 g/L and below [20]. The used concentration is approximately 25 times higher for cement and 20 times higher for bone debris compared to findings in TKA during surgery [21].

In this investigation, for the particle analysis using the acid digestion method [17] and the following SEM analysis, the removal of the added third-bodies was necessary. Thus, the particle lubricant after acid digestion underwent an additional ultracentrifugation step to remove the third-bodies in order to ensure a SEM analysis that just focuses on the generated wear debris. This step is mentioned neither by the ISO nor by the ASTM [16, 22]. This step was successful as no particles in the size range of the third-body debris could be found. On the other hand the loss of particles, especially of the bigger particles, cannot be excluded against the background that over 99% of the particles were sized submicron.

In this test setup a 0.02 *μ*m pore polycarbonate filter membrane was used. An unknown amount of particles below this size might have been lost before the SEM analysis. Currently, it is known that the pore size of the filter could influence the results of the particle analysis. Scott et al. demonstrated that filtration through 0.05 *μ*m is necessary to isolate a greater number of submicron wear particles [23]. With the 0.02 *μ*m pore size filter which was used the majority of the particles should have been isolated.

In general, joint simulators allow preclinical evaluation of wear of artificial joints in a controlled environment [24]. The results of knee simulator studies, in terms of wear volume and size of the debris produced, have been shown to be similar to those found in early retrieved knee prostheses [25–27].

Overall, the particle size distribution in the present study is comparable to those of former particle analyses of bicondylar knee prostheses. As already mentioned, the most particles were found to be submicron, which correlates with the SEM based findings of a knee simulator based study that compared cross-linked and conventional polyethylene inserts for bicondylar knee systems [10]. In this study the mean diameter of the analyzed particles was between 0.37 *μ*m and 0.48 *μ*m and therefore submicron [10]. In the present study the particles are even smaller with 0.13-0.14 *μ*m (given as median) as demonstrated in Table 2. Furthermore, a recent retrieval study by Minoda et al. verified a mean ECD of wear particles from well-functioning total knee prostheses of various material types and designs in a size range from 0.64 *μ*m to 0.81 *μ*m [28]. In this study a filter with a 0.1 *μ*m pore size was used, which might explain the slightly larger values of the particle size distribution.

Interestingly three research groups investigated the impact of methodology concerning a standardized particle analysis in a round robin test [29]. They found that several not exactly defined differences in the complex methodology of wear particle analysis significantly influence the results, for instance, the use of different pore size filter membranes or the use of different SEMs [29]. Therefore, the relatively wide interobserver variability is roughly explainable.

The particles found in the present study were mostly round in shape, smooth, granular, and irregular. According to the sizing of Sieving et al. [13] only the PMMA particle group showed a higher percentage of particles with an AR ≥ 2.4 (13.42%) [13]. This is an important fact, as it is known that fibrillar particles with an AR ≥ 2.4 show increased inflammatory reactions compared to round and granular particles [13, 30]. This has to be assessed with regard to the particle size distribution: Green et al. reported that even small differences in the size range (mean size 0.24 *μ*m versus 0.45 *μ*m and 1.71 *μ*m) lead to different reactions of macrophages* in vitro* [7]. The particles with a mean size of 0.24 *μ*m were the most reactive [7]. As the most particles of all groups in the present study are even smaller it has to be assumed that they are in a biologically reactive size range. The PMMA group, especially, tends towards a higher percentage of fibrillar particles, which are supposed to be biologically more reactive.

Respectively, there are no relevant though statistically significant differences between the groups. The statistical significance is rather due to the extremely high group sizes. This problem occurred in a former study as well [10]; the statistical rating has to be used only carefully.

The most imposing difference between the tested groups in the present study was yet the absolute number of particles per million cycles. The particles were calculated using a previously developed formula [10]. The calculation of the particles is essentially based on two factors: first, the measured gravimetric/volumetric wear rate of the polyethylene inserts and, second, the hypothesized volume of the wear particles. In the calculation of the volume the three-dimensional shape of the particle is assumed to be spherical. Therefore, the ECD of the particles is used as diameter for the calculation. This is an approximately simplified model of the particle shape, as the real volume cannot be assessed using SEM. As the most particles have an AR < 2.39, this assumption seems to be justified [10]. However, the addition of PMMA third-bodies lead to tenfold particle generation compared to the steady state phase and the bone particle group. It is important to note that the number of particles in a given total volume increases as the particle size decreases. This might explain the rather high particle numbers compared to the findings by Utzschneider et al. [10]. They had found particle numbers in the range of 5–20 × 10^9^ [10].

The findings in the present study have to be associated with the complex cellular pathogenesis in the development of periprosthetic osteolysis in response to wear debris [5, 6]. Thus, it has to be assumed that PMMA third-bodies via the generation of multiple particles, especially, negatively influence the biologic reaction and finally lead to an increased inflammatory reaction that ends in an aseptic loosening of the prosthesis. Other factors, including particle surface texture and surface chemistry, could influence the cellular response as well. Further investigation in adequate* in vivo* models is mandatory to clarify the biological activity of the wear debris isolated in this study.

As limitations in the present study the wear simulation tests have to be named. They were performed in a single series of 8.0 million cycles divided in four test groups, rather than different series testing each step. But the advantage in this setup is the identical positioning of the prosthesis throughout the 8.0 million cycles allowing identical test conditions for all groups. Another limitation is the point of time of the addition of the third-bodies. First, they were added after reaching the steady state phase which allows using that phase as a control concerning wear rate and particle generation. This certainly can not totally be transferred to the clinical situation, as the third-bodies are most likely placed already during the implantation of the prosthesis. The order of the third-body particles might influence the results as well. As the wear rate did not change after adding the third-body bone particles compared to the steady state phase, negligible changes of the wear pattern were assumed [12].

The particle analysis was performed using a grayscale detection method. This allows objective particle measurements. On the other hand, small grayscale differences cannot be captured by the software, which might lead to values that do not reflect the absolutely correct size and shape of the particles. Additionally, the geometrical structure of the particles can only be assumed, as SEM analysis does not allow a three-dimensional measurement of the particles.

## 5. Conclusion

The results of the present study demonstrate the evident effect of PMMA third-body wear particles on the particle generation after UKA in a knee simulator based study. The PMMA particles increase the generation of numerous particles and slightly alter the particle morphology towards fibrillar particles. This might lead to an elevated inflammatory response after UKA* in vivo* and, therefore, even lead to an early failure of the unicondylar knee prosthesis.

In this regard the careful removal of PMMA debris and a thorough lavage after UKA implantation is mandatory. In order to detect missed PMMA pieces, postoperative X-ray diagnosis should be used to verify hidden third-bodies.

## Figures and Tables

**Table 1:** Size and shape of third-body particulate debris.

Parameter	Porcine bone particles	PMMA particles
Mean diameter (*µ*m)	671.6 ± 186.4	644.2 ± 262.6
ECD (*µ*m)	519.0 ± 142.9	548.4 ± 237.3
FF	0.41 ± 0.12	0.57 ± 0.12
AR	1.74 ± 0.62	1.74 ± 0.70
Roundness	0.50 ± 0.14	0.42 ± 0.14

**Table 2:** Median size and morphology parameters of all analyzed particles.

	Area (*µ*m^2^)	Roundness	AR	ECD (*µ*m)	FF	Mean diameter (*µ*m)
Bone particles	0.013^*^ (0.003–8.978)	0.674^*^ (0.084–1.176)	1.337^*^ (1.000–5.931)	0.129^*^ (0.062–3.381)	0.680^*^ (0.067–0.903)	0.144^*^ (0.057–5.383)
PMMA particles	0.011^*^ (0.003–8.401)	0.516^*^ (0.067–1.176)	1.509^*^ (1.000–11.862)	0.118^*^ (0.062–3.271)	0.606^*^ (0.035–0.887)	0.130^*^ (0.057–5.742)
Control	0.012^*^ (0.003–6.690)	0.578^*^ (0.062–1.176)	1.509^*^ (1.000–9.897)	0.124^*^ (0.062–2.919)	0.629^*^ (0.033–0.902)	0.130^*^ (0.057–4.995)

^*^ *P* < 0.05*; *in brackets: min.–max.

**Table 3:** Percentage of particles in the distribution described by Sieving et al. [13].

	Aspect ratio	
	1–2.39 (%)	≥2.4 (%)
Bone particles	93.65	6.35
PMMA particles	86.58	13.42
Control	90.16	9.84
